# Ameliorative effect and mechanism of Si-Ni-San on chronic stress-induced diarrhea-irritable bowel syndrome in rats

**DOI:** 10.3389/fphar.2022.940463

**Published:** 2022-08-08

**Authors:** Hui-Yu Chen, Jian Liu, Ding-Zhou Weng, Li Yan, Chun-Shui Pan, Kai Sun, Xiao Guo, Di Wang, Gulinigaer Anwaier, Ying-Qian Jiao, Zhi-Xin Li, Jing-Yan Han

**Affiliations:** ^1^ Department of Integration of Chinese and Western Medicine, School of Basic Medical Sciences, Peking University, Beijing, China; ^2^ Tasly Microcirculation Research Center, Peking University Health Science Center, Beijing, China; ^3^ Academy of Integration of Chinese and Western Medicine, Peking University Health Science Center, Beijing, China; ^4^ Key Laboratory of Microcirculation, State Administration of Traditional Chinese Medicine of the People’s Republic of China, Beijing, China; ^5^ Key Laboratory of Stasis and Phlegm, State Administration of Traditional Chinese Medicine of the People’s Republic of China, Beijing, China; ^6^ State Key Laboratory of Core Technology in Innovative Chinese Medicine, Tianjin, China; ^7^ Beijing Microvascular Institute of Integration of Chinese and Western Medicine, Beijing, China

**Keywords:** Si-Ni-San, sympathetic nerves excitation, energy metabolism, intestinal mucosal barrier, tight junctions

## Abstract

**Background:** Chronic stress-induced diarrhea is a common clinical condition, characterized by an abnormal bowel movement and loose stools, which lacks effective treatment in the clinic. Si-Ni-San (SNS) is a compound traditional Chinese medicine extensively used in China for stress-related diarrhea. However, the mechanism is unclear.

**Methods:** Male Wistar rats (200 ± 20 g) were placed in a restraint cylinder and fixed horizontally for 3 h once daily for 21 consecutive days to establish a chronic restraint stress (CRS) rat model. SNS (0.6944 g/kg or 1.3888 g/kg) was given by gavage 1 h before the restraint once daily for 21 consecutive days. We examined the fecal score, dopamine β hydroxylase (DβH), and c-fos expression in locus coeruleus, norepinephrine (NE) content in ileum and plasma, expression of α1 adrenergic receptors, MLCK, MLC, and p-MLC in the colon and mesenteric arteries, contraction of isolated mesenteric arteries, The expression of subunit δ of ATP synthase (ATP5D) in intestinal tissues, ATP, ADP, and AMP content in the ileum and colon, occludin expression between ileum epithelial cells, the number of enterochromaffin cells (ECs) and mast cells (MCs) in the ileum, and 5-hydroxytryptamine (5-HT) content in the ileum and plasma.

**Results:** After SNS treatment, the fecal score was improved. The increased expression of DβH and c-fos in locus coeruleus was inhibited. SNS suppressed the increased NE content in the ileum and plasma, down-regulated α1 adrenergic receptors in mesenteric arteries and MLCK, MLC, p-MLC in the colon and mesenteric arteries, and inhibited the contraction of mesenteric arteries. SNS also increased the ATP content in the ileum and colon, inhibited low expression of ATP5D in intestinal tissues, inhibited the decrease of ATP/ADP in the ileum and ATP/AMP in the colon, and up-regulated the occludin expression between ileum epithelial cells. In addition, SNS inhibited the increase of ECs and MCs in the ileum and the increase of 5-HT content in the ileum and plasma.

**Conclusion:** This study demonstrated that SNS could improve CRS-induced abnormal feces in rats. This effect was related to the inhibition of CRS-induced increased expression of DβH and c-fos in the locus coeruleus, NE content in the ileum and plasma, and the contraction of isolated mesenteric arteries; inhibition of energy metabolism abnormality and decreased occludin expression; inhibition of increased ECs and MCs in the ileum, and 5-HT content in the ileum and plasma.

## Introduction

Irritable bowel syndrome (IBS) is a chronic functional gastrointestinal disorder, affecting about 11.2% of the global population (Lovell and Ford, 2012; Jun et al., 2022). The prevalence of IBS in North America and Southeastern China is 7.1 and 5.9%, respectively (Long et al., 2017; Sperber et al., 2017; Pei et al., 2020). The annual health care costs for IBS is about eight billion euros in Europe, 123 billion RMB in China, and 10 billion dollars in the United States (Carrasco-Labra et al., 2019; Jiang et al., 2021; Peery et al., 2022). Diarrhea-irritable bowel syndrome (IBS-D) accounts for about 40% of all IBS (Wang et al., 2022) and is the most common type of IBS in China. However, there is no effective treatment for IBS-D in clinics.

Chronic stress-induced diarrhea, characterized by an abnormal bowel movement and loose stools, is a common type of IBS-D (Ek et al., 2015; Chang, 2021). On one hand, chronic stress activates dopamine β hydroxylase (DβH) and up-regulates c-fos in the locus coeruleus, and increases the release of norepinephrine (NE) (Sun et al., 2017). NE could act on the α1D adrenergic receptor in intestinal smooth muscle cells, leading to intestinal contraction, peristalsis, and diarrhea (Kurahashi et al., 2020). On the other hand, chronic stress also impairs the integrity of the intestinal mucosal barrier and increases 5-hydroxytryptamine (5-HT) content (Pohl et al., 2017; Wong et al., 2019). 5-HT is also an important contributor of diarrhea by promoting intestinal peristalsis and mucus secretion (Li et al., 2021).

Si-Ni-San (SNS) is a composed traditional Chinese medicine including Bupleurum (Chaihu), Citrus Aurantium (Zhishi), Radix Glycyrrhizae Preparate (Zhigancao), and Radix Paeoniae Alba (Baishao). SNS is extensively used in China for stress-related diarrhea and clinical evidence showed good efficacy (Li et al., 2019). Bioinformatics studies indicated that the potential mechanism may involve the inhibition of dopaminergic synapses and the activation of amphetamine addiction signaling pathways (Li et al., 2019). Furthermore, SNS also improved function dyspepsia partially through restoring tight junctions and the mucosal barrier’s integrity in the duodenum of the rats (Chang et al., 2017; Zhu et al., 2020). However, whether SNS could improve IBS-D and the underlying mechanism is not clear.

The present study aims to investigate the ameliorative effects and mechanisms of Si-Ni-San on chronic stress-induced IBS-D in rats with a special focus on DβH and c-fos activation in the locus coeruleus, NE, and 5-HT content in the intestine, α1 adrenergic receptor, and its down-stream signaling in the intestine, and the intestinal mucosal barrier’s integrity.

## Material and methods

### Reagents and antibodies

SNS granules were composed of Bupleurum (Chaihu) granules (25%), Radix Paeoniae Alba (Baishao) granules (25%), Citrus Aurantium (Zhishi) granules (25%), and Radix Glycyrrhizae Preparate (Zhigancao) granules (25%), which were purchased from Yifang Pharmaceuticals (Guangdong, China). The primary antibodies against c-fos, CD31, mast cell tryptase, 5-HT, occludin, and MLCK were purchased from Abcam (Cambridge, UK). An anti- DβH antibody was purchased from Thermo Scientific (Rockford, Illinois, United States). NE, 5-HT, ATP, ADP, and AMP ELISA kits were purchased from Andy Gene Biotechnology (Beijing, China).

### Animals

Male Wistar rats (200 ± 20 g) were obtained from Vital River Laboratory Animal Technology (Beijing, China, certificate number SCXK 2016-0011). The rats were kept at a temperature of 20 ± 2 °C and a humidity of 40 ± 5% with 12/12 h light/dark cycles, and fed with a standard laboratory diet and water. Animal care was in accordance with the guidelines of Peking University Animal Research Committee. The experimental protocols were approved by the Committee on the Ethics of Animal Experiments of Peking University Health Science Center (LA2021360) and were in accordance with the Guide for the Care and Use of Laboratory Animals published by the National Institute of Health (eighth edition, 2011).

### CRS protocol and stool evaluation

To establish the CRS model (Julio-Pieper et al., 2012), the rats were placed in a restraint cylinder and fixed horizontally for 3 h once daily for 21 consecutive days. During each restraint stress period, the occurrence of diarrhea was assessed using an arbitrary scoring scale (Kawano et al., 2005) ranging from 0 to 3: 0, absence of fecal pellets; 1, well formed, solid pellets; 2, slightly wet and soft to touch but formed pellets; and 3, watery and unformed stools. The body weight and food intake of the rats were recorded every 48 h 2 h after the last restraint stress, the rats were sacrificed, and tissue and plasma samples were collected.

### Experimental groups and drug treatment

A total of 52 rats were randomly divided into four groups, 13 rats in each group: 1) Control group, 2) CRS + normal saline (NS) group, 3) CRS + SNS (0.6944 g/kg) group, and 4) CRS + SNS (1.3888 g/kg) group. The number of rats used for each parameter is also listed in Table 1. The low-dose SNS used in this study (0.6944 g/kg in rats) is equivalent to the clinical dose in humans, and the high-dose SNS (1.3888 g/kg) is 2-times the clinical dose in humans. The dosage of SNS for a human is 0.744 g herbal medicine/kg body weight daily. That is, 5.208 g herbal medicine/kg/day in rats, which equals to 0.6944 g SNS formula granule/kg/day according to the manufacturer’s instruction. All the treatments were given by gavage 1 h before restraint at a volume of 10 ml/kg once daily for 21 consecutive days.

**TABLE 1:** Number of rats in each group and used in each parameter.

Parameter/Group	Control	CRS + NS	CRS + SNS (0.6944 g/kg)	CRS + SNS (1.3888 g/kg)	Total
**Weight/Food-intake**	**8**	**8**	**8**	**8**	**32**
**Molecular biology**	**(4-6)**	**(4-6)**	**(4-6)**	**(4-6)**	**(12-24)**
**Mesenteric arteriole tension measurement**	**(6)**	**(6)**	**(6)**	**(6)**	**(24)**
**Stool score evaluation**	**5**	**5**	**5**	**5**	**20**
**Histological staining**	**(3)**	**(3)**	**(3)**	**(3)**	**(12)**
**Total**	**13**	**13**	**13**	**13**	**52**

Bracketed numbers indicated that the samples used for Molecular biology and Mesenteric arteriole tension measurement were from the eight rats used for Weight/Food-intake, and the samples used for Histological staining were from the five rats used for Stool score evaluation. The bold values 0.6944 g/kg and 1.3888 g/kg indicates the dosages of the drug used in rats and the bold numbers are represents the number of rats used in each group or for each parameter.

### ELISA assay

The levels of NE, 5-HT in plasma, ileum tissue, and ATP, ADP, AMP in the ileum and colon tissues were all detected by ELISA kits according to the manufacturer’s instructions (Andy Gene, Beijing, China).

### Hematoxylin-eosin (HE) staining

The ileum and colon were harvested and fixed in 4% paraformaldehyde and processed for paraffin sectioning (5 µm) using an Automatic Paraffin slicer (Leica 2M2255, Leica, Mannheim, Germany). The sections were stained with H and E and scanned by a digital slide scanner (3DHISTECH, Budapest, Hungary).

### Immunohistochemistry staining

After dewaxing and antigen retrieval, the slides were incubated with 0.3% Triton X-100 for 30 min, and then with hydrogen peroxide for 10 min at room temperature. The slides were washed with PBST and blocked with goat serum for 30 min, and incubated with primary antibodies against mast cell tryptase (1:100) and 5-HT (1:100) overnight at 4 °C. Then, specific binding was detected by incubating with an HRP-conjugated secondary antibody and positive staining was visualized with a DAB substrate kit. Images were taken using a digital slide scanner (3DHISTECH, Budapest, Hungary).

### Immunofluorescence staining

After dewaxing, antigen retrieval, and blocking, the sections were incubated with primary antibodies against anti-DβH (1:50, Thermo Scientific, Rockford, Illinois, United States), c-fos (1:100, Abcam, Cambridge, UK), occludin (1:150, Abcam, Cambridge, UK), keratin (1:50, Cell Signaling Technology, Massachusetts,United States), and CD31 (1:150, Abcam, Cambridge, UK), α-SMA (1:150, Abcam, Cambridge, UK) antibodies and incubated overnight at 4 °C. Then, the sections were incubated with a fluorescent secondary antibody (1:100) for 2 h. A laser scanning confocal microscope (TCS STED, Leica, Mannheim, Germany) was used to detect positive staining.

### Western blotting analysis

The rats were sacrificed, and the ileum, colon, and brain tissues were collected and frozen to −80 C. The total protein was extracted using a protein extraction kit (Applygen Technologies, Beijing, China) and then was separated by sodium dodecyl sulfate-polyacrylamide gel electrophoresis and transferred to a polyvinylidene fluoride (PVDF) membrane. After blocking with 5% bovine serum albumin, the membrane was incubated overnight at 4 °C with antibodies against DβH (1:1000, Thermo Scientific, Rockford, Illinois, United States), c-fos (1:1000, Abcam, Cambridge, UK), MLCK (1:1000, ImmunoWay Biotechnology Company, Plano, United States), MLC (1:1000, Cell Signaling Technology, Massachusetts,United States), p-MLC (1:1000, Cell Signaling Technology, Massachusetts,United States), GAPDH (1:2000, Cell Signaling Technology, Massachusetts,United States), and α1-ARs (1:1000, Proteintech, Rosemont, United States). After washing, the membrane was incubated with a secondary antibody for 1 h at room temperature and then the immunoreactive bands were revealed using an enhanced chemiluminescence system and analyzed using the Quantity One image analyzer software (Bio-Rad, Texas, United States).

### *Ex vivo* mesenteric arteriole tension measurement

After the rats were anesthetized, the mesenteric arterioles were isolated, and cut into rings about 2.0 mm in length. The arteriole rings were suspended in a wire myograph (Danish Myo Technology, Denmark) for measuring SNS induced vaso-relaxation and NE-induced vaso-contraction as previously reported (Zhang et al., 2018).

### Statistical analysis

All results were presented as mean ± S.E.M. Body weight and food-intake were analyzed using two-way ANOVA followed by Bonferroin, and the remaining results were analyzed using one-way ANOVA followed by a Newman–Keuls test. The data were analyzed using the GraphPad Prism nine software (GraphPad software Inc., CA, United States). *P* value less than 0.05 was considered to be statistically significant.

## Results

### SNS improved the fecal score induced by CRS

**FIGURE 1:**
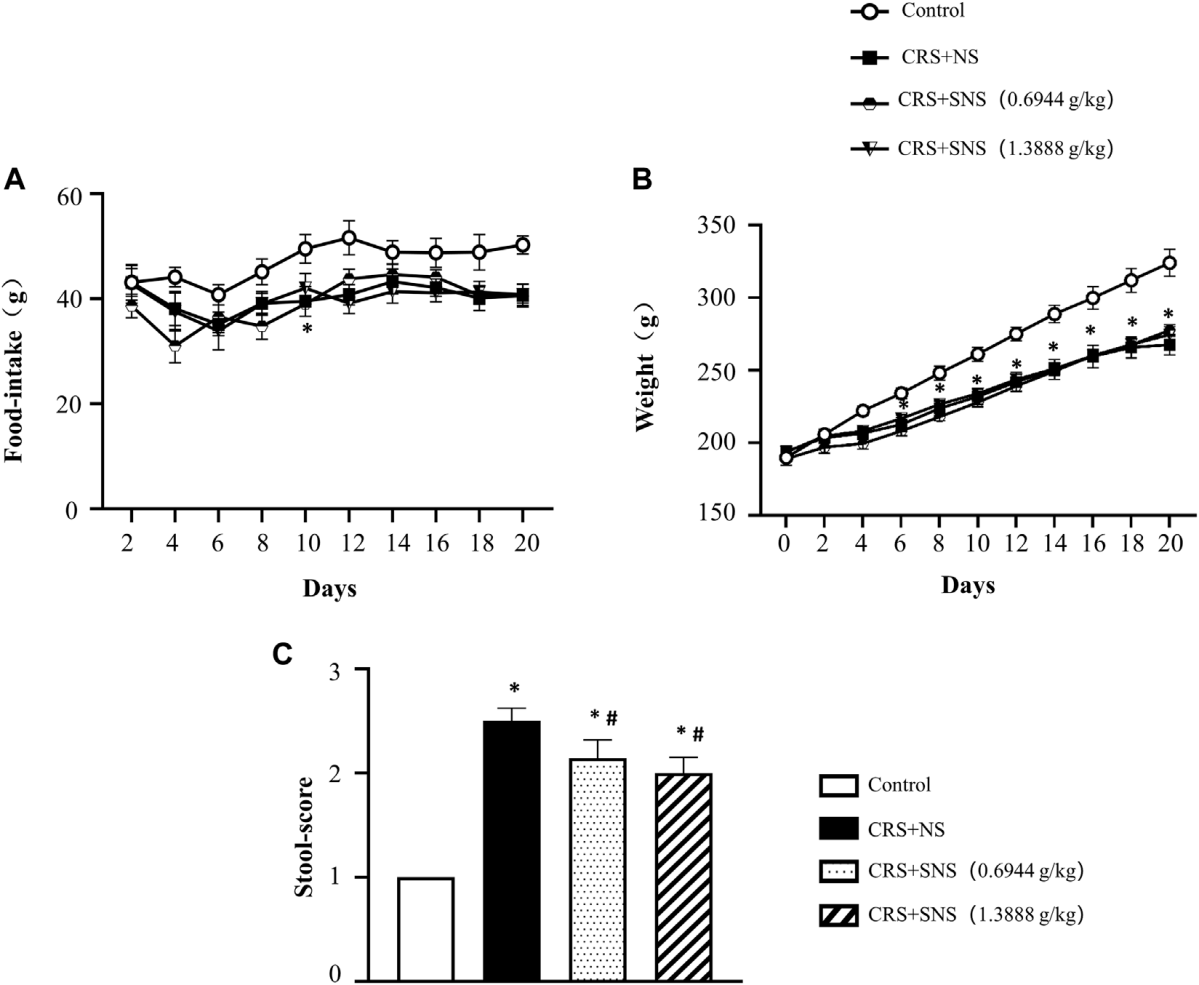
Effect of the SNS treatment on rat weight, food-intake, and stools. **(A)** The changes in the food-intake in different groups. F = 11.99. *p* < 0.0001. **(B)** The changes in the body weight in different groups. F = 1.967. *p* = 0.1345. **(C)** The fecal score of different groups. F = 112.7. *p* < 0.0001. Results are presented as mean ± SEM.*p < 0.05 vs. Control group; #*p* < 0.05 vs. CRS + NS group. **(A,B)** *n* = 8. **(C)** *n* = 5.

The changes in the body weight and food intake of the rats were recorded. As shown in Figures 1A, B, compared with the Control group, the food intake and body weight of the rats in the CRS + NS group were significantly reduced. SNS treatment had no effect on the food intake and body weight. The fecal score of the CRS + NS group was significantly higher than the Control group, indicating more watery and unformed stools induced by CRS, while SNS treatment significantly reduced the fecal score and improved diarrhea symptoms (Figure 1C).

### SNS inhibited increased DβH and c-fos expressions in the locus coeruleus induced by CRS

**FIGURE 2:**
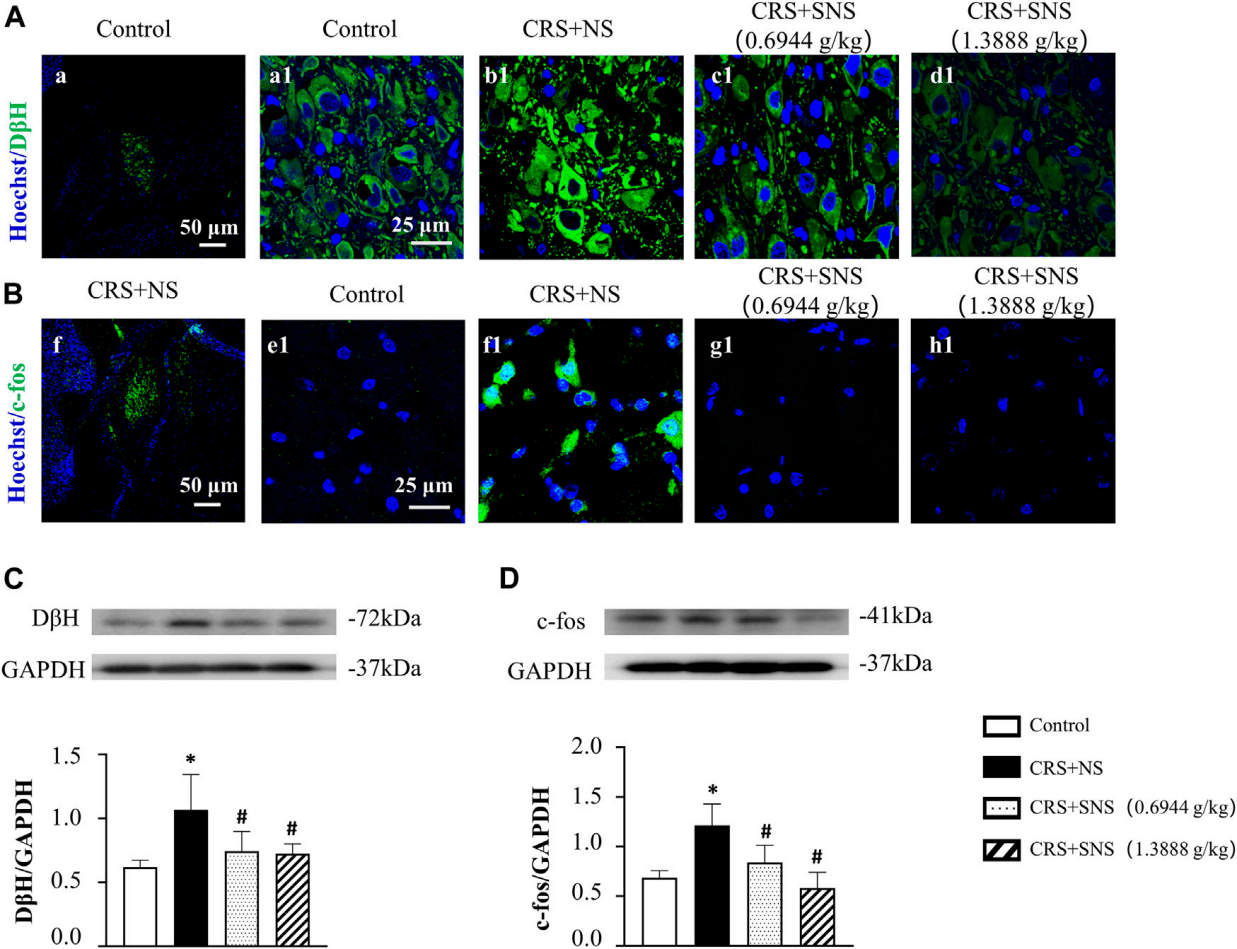
Effect of the SNS treatment on the expression of DβH and c-fos in locus coeruleus. **(A)** Representative immunofluorescent images of DβH (green) and nuclei (blue) in the Control (a1), CRS + NS (b1), CRS + SNS (0.6944 g/kg) (c1), and CRS + SNS (1.3888 g/kg) (d1) groups, respectively. Bar = 25 μm. **(A)** is the image of DβH in the Control group in a low-power lens. Bar = 50 μm. **(B)** Representative immunofluorescent images of c-fos (green) and nuclei (blue) in the Control (e1), CRS + NS (f1), CRS + SNS (0.6944 g/kg) (g1), and CRS + SNS (1.3888 g/kg) (h1) groups, respectively. Bar = 25 μm. **(F)** is the image of c-fos in the CRS + NS group in a low-power lens. Bar = 50 μm. **(C)** Western blot of DβH in the locus coeruleus of different groups with quantification showing below. F = 6.178. *p* = 0.0054. **(D)** Western blot of c-fos in locus coeruleus of different groups with quantification showing below. F = 11.94. *p* = 0.0002. Results are presented as mean ± SEM. *p < 0.05 vs. Control group; #*p* < 0.05 vs. CRS + NS group. **(A,B)** n = 3. **(C,D)** *n* = 5.

In our previous study (Sun et al., 2017), we proved that chronic unpredictable mild stress could cause locus coeruleus activation, DβH and c-fos up-regulations, and an increased release of NE. We thus detected DβH and c-fos in the locus coeruleus by immunofluorescence and Western blotting analysis. The result of the immunofluorescence showed that, the expression of DβH (Figure 2A) and c-fos (Figure 2B) increased significantly in the CRS + NS group compared with the Control group, which was blocked by SNS treatment. The result from the Western blot also showed that SNS treatment inhibited CRS-induced DβH and c-fos up-regulations (Figures 2C, D). These results indicated the potential of SNS to regulate the activity of the sympathetic nervous system.

### SNS suppressed the increase of NE content and MLCK, p-MLC and MLC expressions in the colon induced by CRS

**FIGURE 3:**
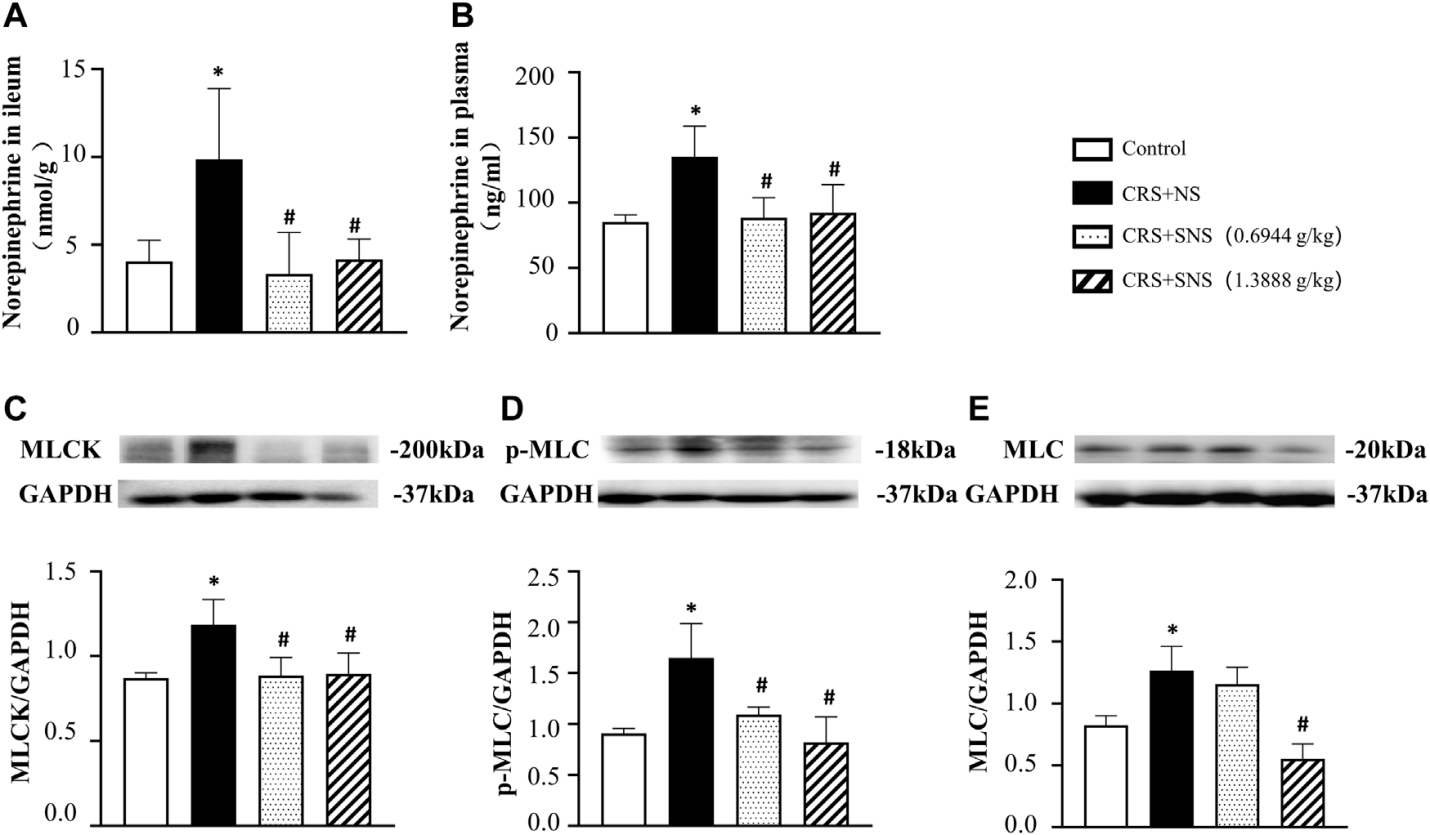
Effect of the SNS treatment on the level of NE in the ileum tissue and plasma and the expression of MLCK, p-MLC, and MLC in the colon tissue. **(A)** NE concentration in the ileum tissue from different groups. F = 6.027. *p* = 0.0060. **(B)** NE concentration in plasma from different groups. F = 6.458. *p* = 0.0075. **(C)** Western blot of MLCK in the colon tissue from different groups with quantification showing below. F = 6.947. *p* = 0.0058. **(D)** Western blot of p-MLC in colon tissue from different groups with quantification showing below. F = 10.83. *p* = 0.0010. **(E)** Western blot of MLC in colon tissue from different groups with quantification showing below. F = 17.71. *p* = 0.0001. Results are presented as mean ± SEM. *p < 0.05 vs. Control group; #*p* < 0.05 vs. CRS + NS group. **(A)** *n* = 5. **(B–E)** *n* = 4.

To study the effect of SNS on the release of NE, we measured the level of NE in the rats’ ileum tissue and plasma. As shown in Figures 3A, B, the level of NE in the ileum tissue and plasma increased significantly after CRS. Treatment with SNS suppressed the aforementioned changes. NE can activate the α1D adrenergic receptors and its downstream MLCK-MLC signaling pathway in gastrointestinal smooth muscle (Castellani and Young, 2016; Kurahashi et al., 2020). As shown in Figures 3C–E, the levels of MLCK, p-MLC, and MLC in the colon tissue of the rats in the CRS + NS group were significantly up-regulated compared to the Control group. Both low-dose and high-dose SNS treatment can inhibit CRS-induced MLCK and p-MLC up-regulations (Figures 3C, D), while only a high-dose SNS treatment can inhibit CRS-induced MLC up-regulation (Figure 3E).

### SNS inhibited intestinal artery contraction induced by CRS

**FIGURE 4:**
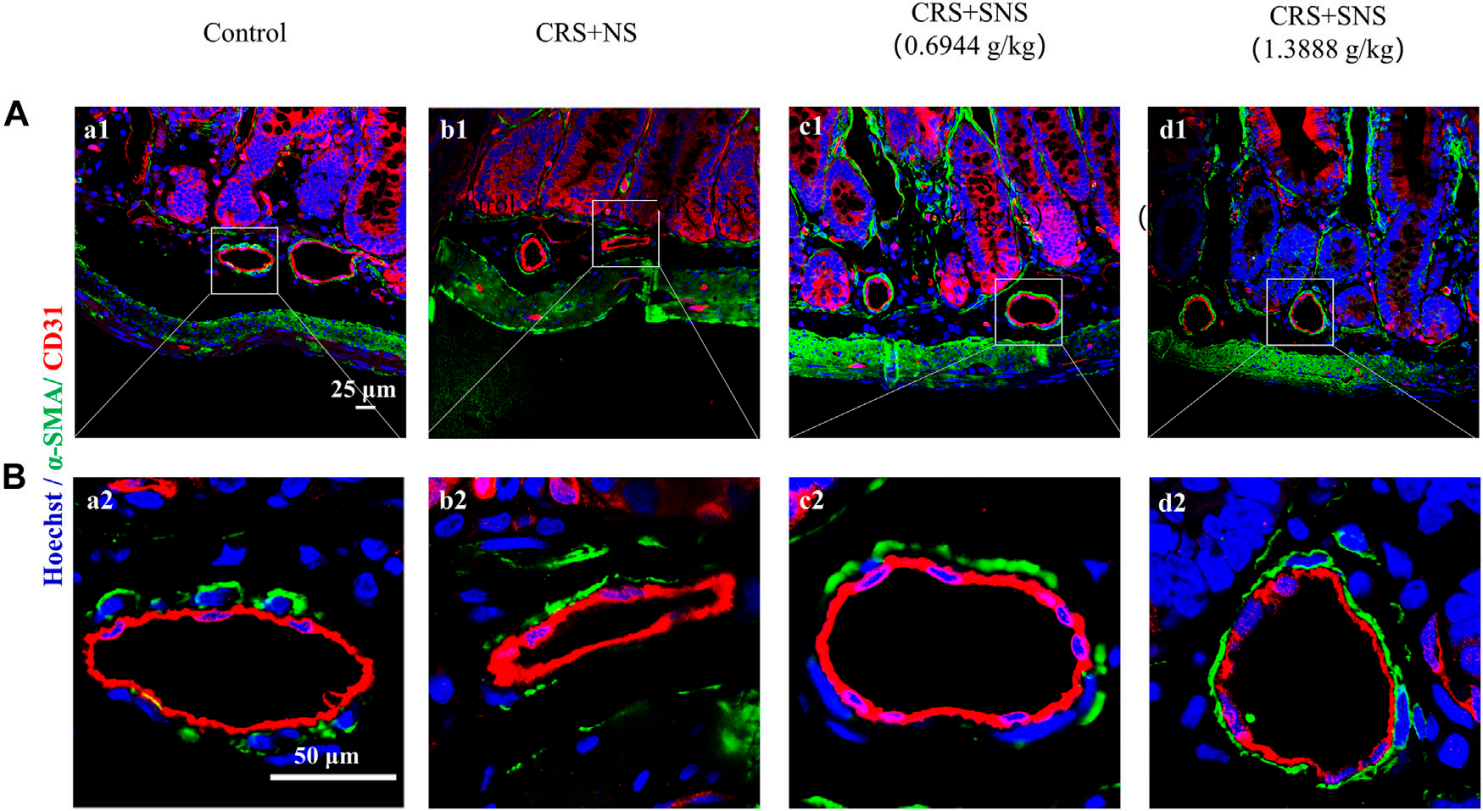
Effect of the SNS treatment on the morphological changes of small arteries in the ileum. **(A)** Representative low magnification of immunofluorescent images of small arteries in the ileum in Control (a1), CRS + NS (b1), CRS + SNS (0.6944 g/kg) (c1), CRS + SNS (1.3888 g/kg) (d1), respectively. Bar = 25 μm. **(B)** Representative high magnification of the immunofluorescent images of small arteries in the ileum in the Control (a2), CRS + NS (b2), CRS + SNS (0.6944 g/kg) (c2), and CRS + SNS (1.3888 g/kg) (d2) groups, respectively. Bar = 50 μm. The sections were immunochemically stained for α-SMA (green), CD31 (red), and nuclei (blue). **(A,B)** *n* = 3.

Excessive NE acting on the intestinal arteries will cause vaso-contraction. Indeed, the small arteries in the ileum of the CRS + NS group were wrinkled and the vessel lumens were narrowed as shown by the immunofluorescence staining (Figures 4A, B). However, this change was not seen in the low-dose and high-dose SNS treatment groups, suggesting that the SNS treatment inhibited the contraction of the small arteries (Figures 4A, B).

**FIGURE 5:**
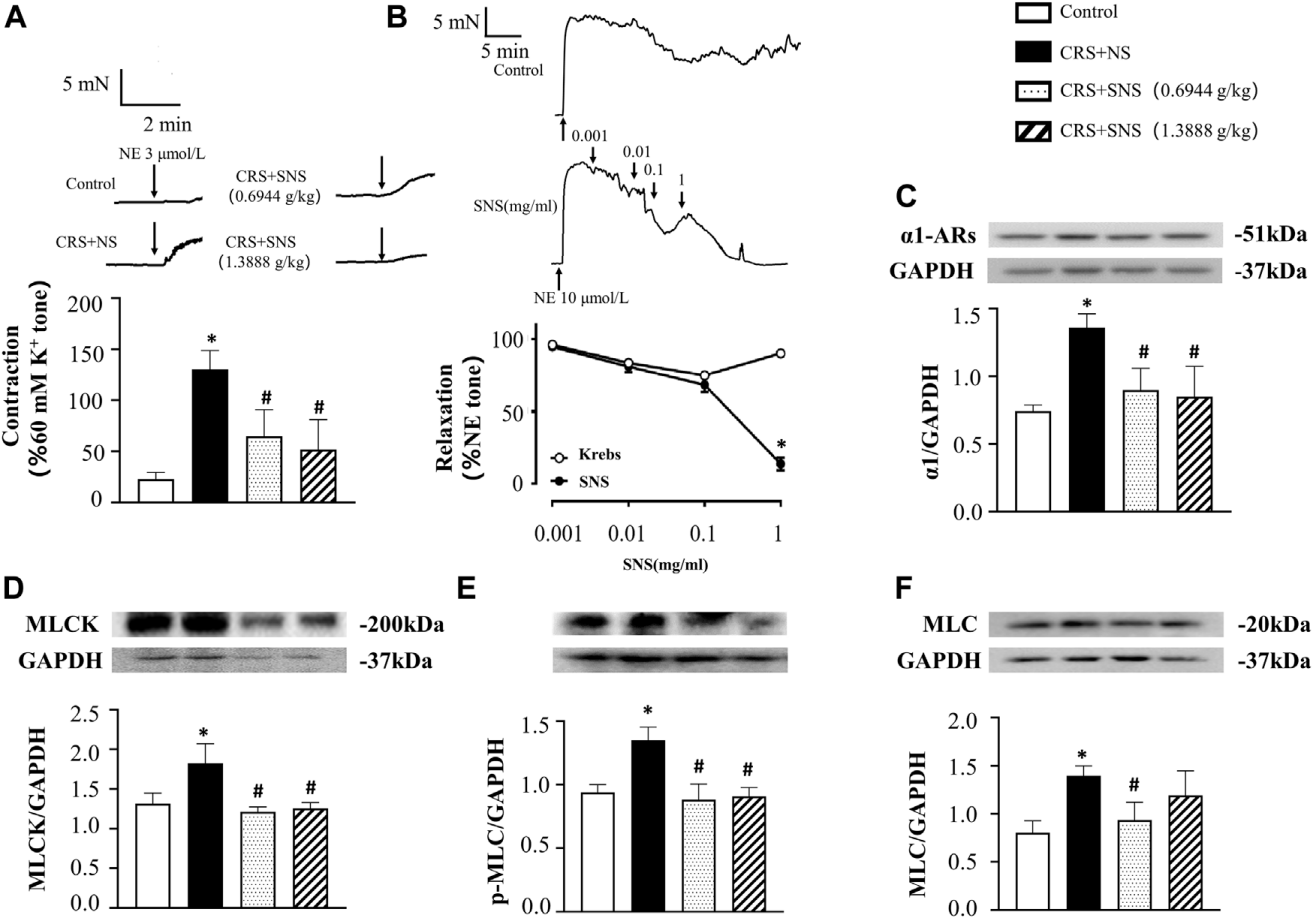
Effect of the SNS treatment on the contraction and expression of α1-ARs, MLCK, p-MLC, and MLC in mesenteric arteries. **(A)** 3 μmol/L NE-induced contraction in the rat mesenteric arteries. F = 23.36. *p* < 0.0001. **(B)** SNS-induced relaxation in mesenteric arteries. **(C)** Western blot of α1-ARs in mesenteric arteries from different groups with the quantification shown below. F = 12.49. *p* = 0.0005. **(D)** Western blot of MLCK in mesenteric arteries from different groups with the quantification shown below. F = 9.030. *p* = 0.0021. **(E)** Western blot of p-MLC in the mesenteric arteries from different groups with the quantification shown below. **(F)** = 17.28. *p* = 0.0001. **(F)** Western blot of MLC in the mesenteric arteries from different groups with quantification showing below. F = 6.582. *p* = 0.0070. Results are presented as mean ± SEM. *p < 0.05 vs. Control group; #*p* < 0.05 vs. CRS + NS group. **(A,B)** *n* = 6. **(C–F)** *n* = 4.

Figure 5A shows the result of NE-induced contraction in rat mesenteric arteries *ex vivo*. Compared with the Control group, NE induced a higher contraction in the arteries of the CRS + NS group. Both low-dose and high-dose SNS treatment groups showed less contraction in response to NE compared with the CRS + NS group. Furthermore, in the mesenteric arteries already contracted by NE, SNS dose-dependently induced vasorelaxation (Figure 5B). The changes in α1-ARs and its downstream MLCK-MLC pathway were also examined. As shown in Figures 5C–F, the expression of α1-ARs, MLCK, p-MLC, and MLC in mesenteric arteries of the CRS + NS group increased significantly. Both low-dose and high-dose SNS treatments inhibited the increased expression of α1-ARs, MLCK, and p-MLC in mesenteric artery tissues (Figures 5C–E), while only the low-dose SNS treatment inhibited the increased expression of MLC (Figure 5F). These results may explain the inhibitory effect of SNS treatment on NE-induced contraction in rats’ intestinal and mesenteric arteries.

### SNS restored the energy metabolism and intestinal barrier integrity

**FIGURE 6:**
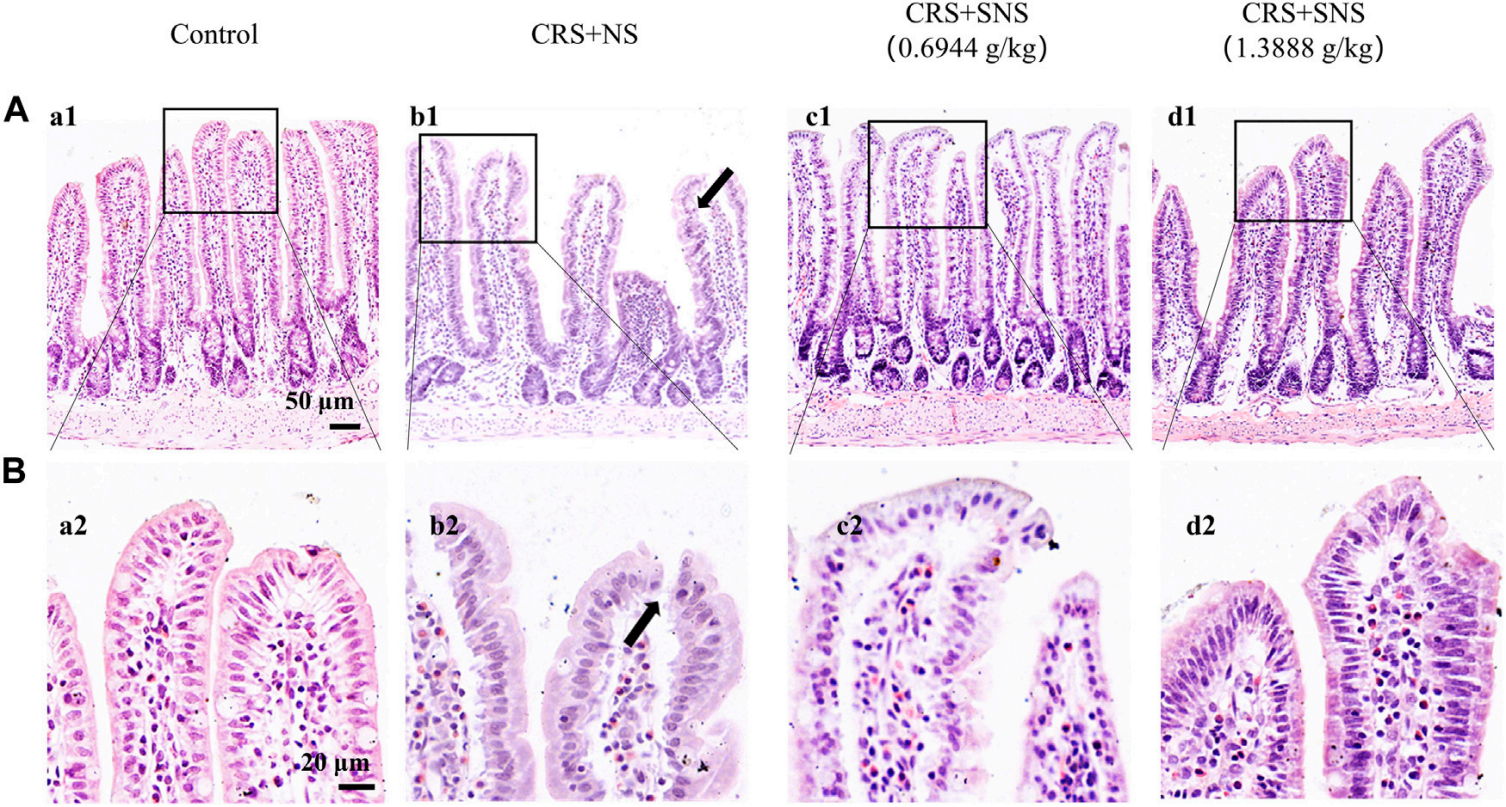
Effect of the SNS treatment on morphological changes of the ileum. **(A)** Representative low magnification of HE staining images of the ileum in the Control (a1), CRS + NS (b1), CRS + SNS(0.6944 g/kg) (c1), and CRS + SNS (1.3888 g/kg) (d1) groups, respectively. Bar = 50 μm. **(B)** Representative high magnification of HE-staining images of the ileum in the Control (a2), CRS + NS (b2), CRS + SNS(0.6944 g/kg) (c2), CRS + SNS (1.3888 g/kg) (d2) groups, respectively. Bar = 20 μm. **(A,B)** *n* = 3.

**FIGURE 7:**
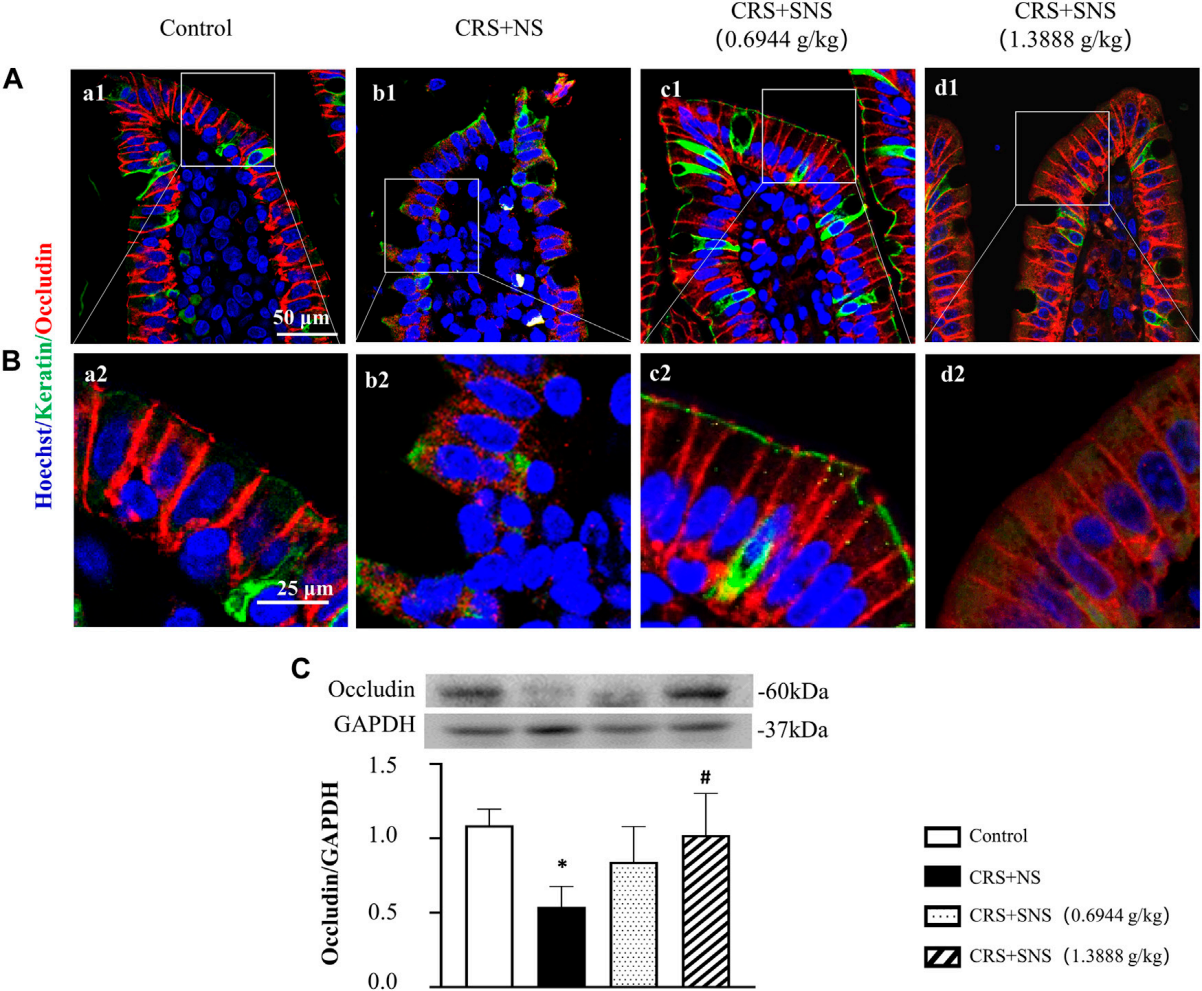
Effect of the SNS treatment on the occludin expression in the ileum epithelium. **(A)** Representative low magnification of immunofluorescent staining images of ileum epithelium in Control (a1), CRS + NS (b1), CRS + SNS(0.6944 g/kg) (c1), CRS + SNS (1.3888 g/kg) (d1), respectively. The sections were immunochemically stained for Keratin (green), occludin (red), and nuclei (blue). Bar = 50 μm. **(B)** Representative high magnification of immunofluorescent staining images of the ileum epithelium in the Control (a2), CRS + NS (b2), CRS + SNS(0.6944 g/kg) (c2), CRS + SNS (1.3888 g/kg) (d2) groups, respectively. The sections were immunochemically stained for Keratin (green), Occludin (red), and nuclei (blue). Bar = 25 μm. **(C)** Western blot of the occludin in the ileum of different groups with quantification showing below. F = 6.376. *p* = 0.0033. Results are presented as mean ± SEM. *p < 0.05 vs. Control group. **(A,B)** *n* = 3. **(C)** *n* = 6.

**FIGURE 8:**
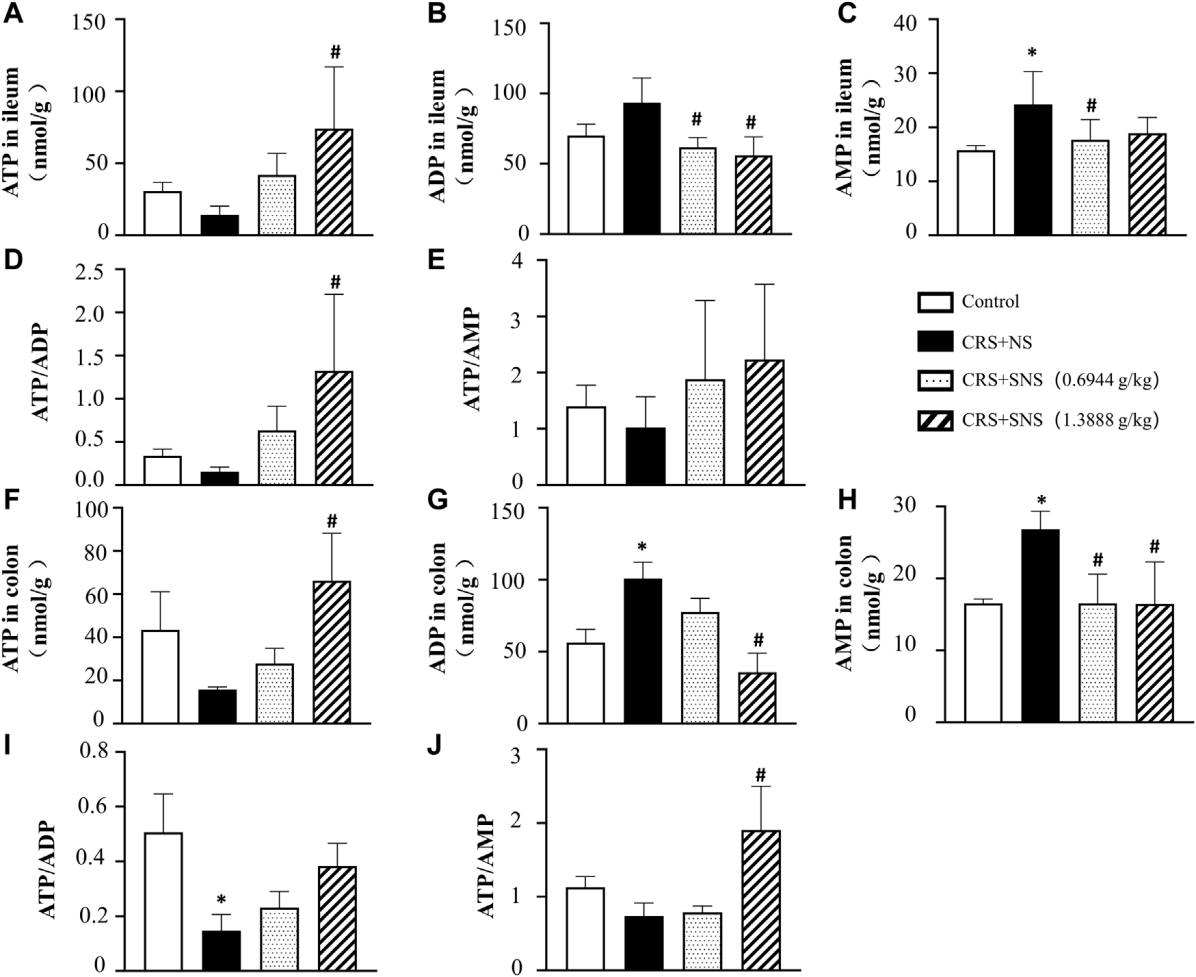
Effect of the SNS treatment on the levels of ATP, ADP, and AMP in the ileum and colon tissues. **(A)** ATP concentration in the ileum tissues from different groups. F = 4.553. *p* = 0.0237. **(B)** ADP concentration in ileum tissues from different groups. F = 6.993. *p* = 0.0021. **(C)** AMP concentration in ileum tissues from different groups. F = 4.918. *p* = 0.0102. **(D)** ATP/ADP in ileum tissues from different groups. F = 4.760. *p* = 0.0207. **(E)** ATP/AMP in ileum tissues from different groups. F = 1.394. *p* = 0.2738. **(F)** ATP concentration in colon tissues from different groups. F = 4.228. *p* = 0.0295. **(G)** ADP concentration in colon tissues from different groups. F = 17.38. *p* = 0.0001. **(H)** AMP concentration in colon tissues from different groups. F = 7.342. *p* = 0.0047. **(I)** ATP/ADP in colon tissues from different groups. F = 4.419. *p* = 0.0259. **(J)** ATP/AMP in colon tissues from different groups. F = 9.628. *p* = 0.0016. Results are presented as mean ± SEM. *p < 0.05 vs. Control group; #*p* < 0.05 vs. CRS + NS group, **(B,C,E)** *n* = 6. **((A,D,F–J)** *n* = 4.

**FIGURE 9:**
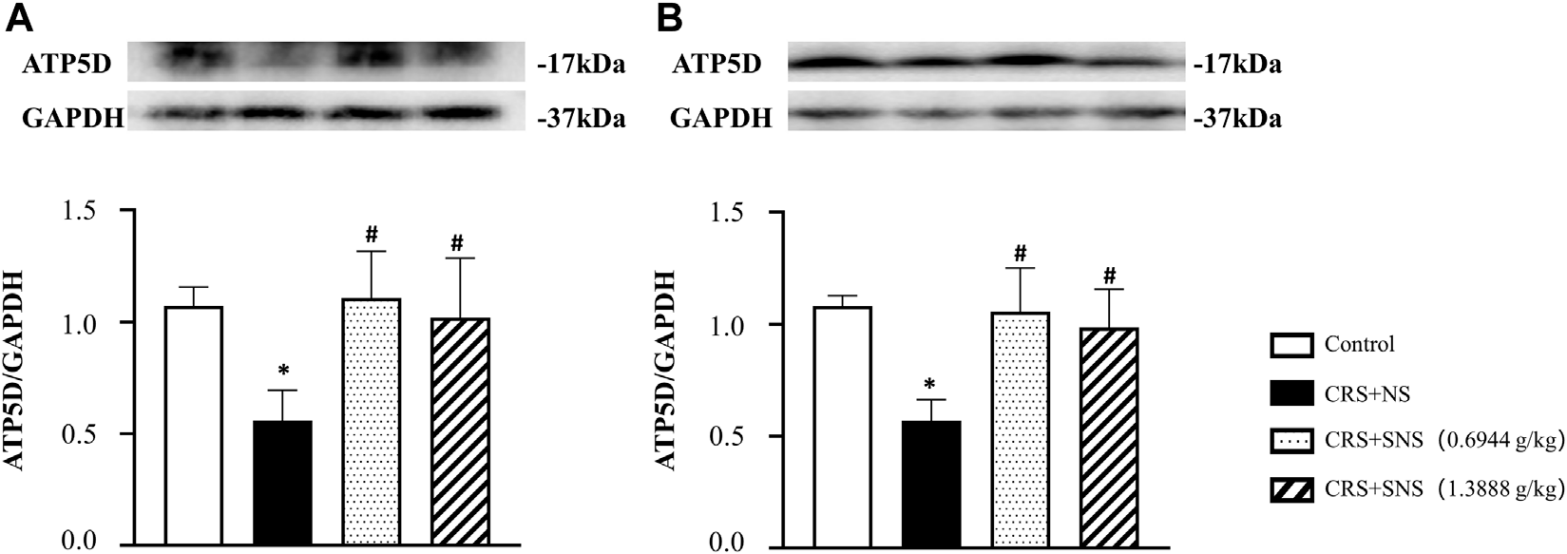
Effect of the SNS treatment on the expression of ATP5D in the ileum and colon tissues. **(A)** Western blot of ATP5D in ileum tissues from different groups with quantification showing below. F = 6.504. *p* = 0073. **(B)** Western blot of ATP5D in colon tissues from different groups with quantification showing below. F = 10.96. *p* = 0.0009. Results are presented as mean ± SEM. *p < 0.05 vs. Control group; #*p* < 0.05 vs. CRS + NS group. **(A,B)** *n* = 4.

As shown in Figures 6A, B, the intestinal villi were sparse and short, and the edema was found around the microvascular in the villi in the CRS + NS group, which was partially prevented by the low-dose and high-dose SNS treatments. At the same time, the expression and distribution of occludin were analyzed by immunofluorescence and Western blot. As shown in Figures 7A, B, compared with the Control group, the distribution of occludin in the CRS + NS group was disorganized and the expression was low. Evidently, treatment with SNS significantly prevented the reduction in the expression and disorganized distribution of occludin between ileum epithelial cells. A similar trend was also found in the result of the Western blotting analysis (Figure 7C). Thus, SNS can improve the intestinal mucosal barrier integrity by restoring the tight junctions (TJs) proteins of the intestinal epithelium. Our previous study showed that an abnormal energy metabolism would break endothelial TJs (Han et al., 2017). To further investigate the mechanism by which SNS improved intestinal epithelial TJs, we used ELISA to detect the level of ATP, ADP, and AMP in the ileum and colon tissue. Compared with the Control group, CRS did not change the level of ATP, but increased ADP in the colon and AMP in both the ileum and colon; low-dose SNS treatment did not change the level of ATP, but significantly decreased the level of ADP in the ileum and AMP in both the ileum and colon; high-dose SNS significantly increased the level of ATP and decreased the level of ADP in both the ileum and colon, and decreased the level of AMP in the colon (Figures 8A–C, F–H). Additional, we also found CRS can decrease the ATP5D expression in ileum and colon tissues, both low-dose and high-dose SNS treatments inhibited the decreased expression of ATP5D in the ileum and colon tissues (Figures 9A, B).

### SNS inhibited the increase of 5-HT in the ileum tissue and plasma and ECs in the ileum tissue induced by CRS

**FIGURE 10:**
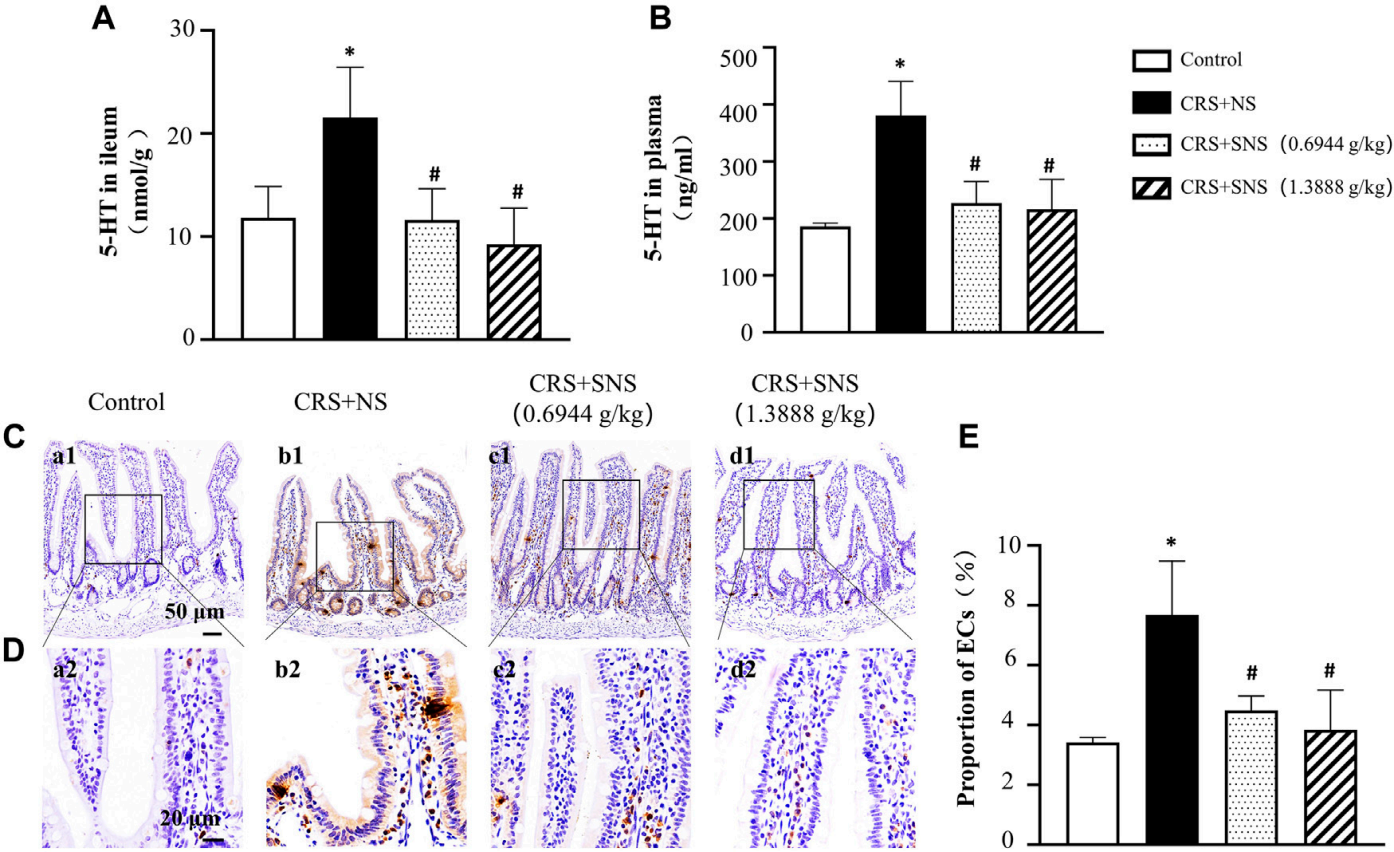
Effect of the SNS treatment on 5-HT content in the ileum tissue and plasma and on the number of ECs of the ileum. **(A)** 5-HT concentration in ileum tissues from different groups. F = 5.845. *p* = 0.0106. **(B)** 5-HT concentration in the plasma from different groups. F = 15.30. *p* = 0.0002. **(C)** Representative low magnification of immunehistochemical staining images of anti-5-HT antibody in the ileum tissues in Control (a1), CRS + NS (b1), CRS + SNS (0.6944 g/kg) (c1), CRS + SNS (1.3888 g/kg) (d1) groups, respectively. Bar = 50 μm. **(D)** Representative high magnification of immunohistochemical staining images of the anti-5-HT antibody in the ileum tissues in the Control (a2), CRS + NS (b2), CRS + SNS(0.6944 g/kg) (c2), CRS + SNS (1.3888 g/kg) (d2) groups, respectively. Bar = 20 μm. **(E)** The counts of ECs in the ileum tissue of the rats in each group. F = 8.487. *p* = 0072. Results are presented as mean ± SEM.*p < 0.05 vs. Control group; #*p* < 0.05 vs. CRS + NS group. **(A,B)** *n* = 4. **(C–E)** *n* = 3.

5-HT is involved in chronic stress-induced diarrhea (Gao et al., 2022). In order to study the effect of SNS on 5-HT secretion induced by CRS, the levels of 5-HT in the ileum tissue and plasma were detected. As shown in Figures 10A, B, compared with the Control group, the levels of 5-HT in the plasma and ileum tissues increased significantly after CRS, which were diminished in the SNS treatment groups. As more than 90% of the 5-HT were secreted by ECs (Gershon, 2013; Linan-Rico et al., 2016; Bellono et al., 2017), we then detected ECs in the ileum by immunochemistry staining of 5-HT. Consistent with the 5-HT levels, the number of ECs in the ileum tissue in CRS + NS group was higher than in the Control group (Figures 10C–E), which was again ameliorated by SNS treatment.

### SNS inhibited the increase of MCs in the ileum tissue induced by CRS

**FIGURE 11:**
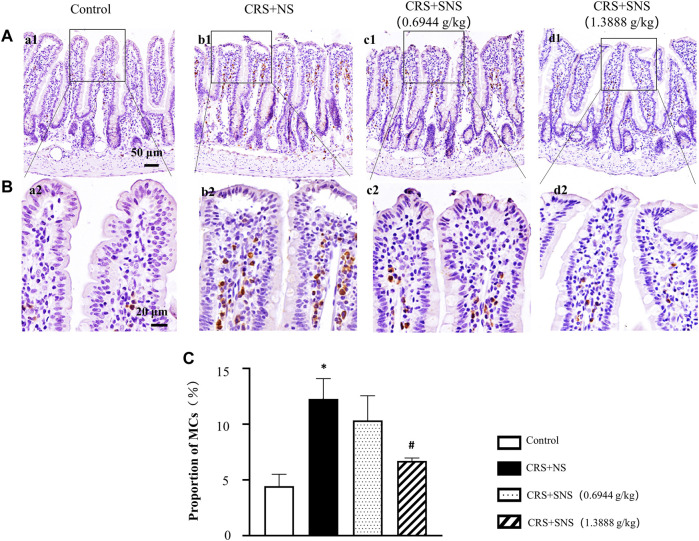
Effect of the SNS treatment on the number of MCs of ileum. **(A)** Representative low magnification of immunohistochemical staining images of the anti-tryptase antibody in ileum tissues in the Control (a1), CRS + NS (b1), CRS + SNS(0.6944 g/kg) (c1), CRS + SNS (1.3888 g/kg) (d1) groups, respectively. Bar = 50 μm. **(B)** Representative high magnification of immunohistochemical staining images of the anti-tryptase antibody in ileum tissues in the Control (a2), CRS + NS (b2), CRS + SNS(0.6944 g/kg) (c2), CRS + SNS (1.3888 g/kg) (d2) groups, respectively. Bar = 20 μm. **(C)** The counts of MCs in the ileum tissues of the rats in each group. F = 12.82. *p* = 0.0020. Results are presented as mean ± SEM. *p < 0.05 vs. Control group; #*p* < 0.05 vs. CRS + NS group. **(A–C)** *n* = 3.

Previous studies have reported that increased MCs were associated with intestinal permeability and diarrhea (Aguilera-Lizarraga et al., 2022). Tryptase is an important marker of MCs; therefore, the effect of SNS on MC infiltration was assessed by the immunochemistry staining of tryptase. As shown in Figures 11A–C, compared with the Control group, the number of MCs in the ileum of CRS + NS group was significantly increased. Only the high-dose and not the low-dose SNS inhibited the increase in the number of MCs.

## Discussion

This study demonstrated that SNS could improve CRS-induced diarrhea in rats by inhibiting the CRS-induced up-regulation of DβH and c-fos in the rat brain locus coeruleus, suppressing the increased intestinal and plasma NE content, down-regulating α1-ARs, MLCK, MLC, and p-MLC, and inhibiting the contraction of small arteries in the intestine. The SNS treatment also increased the ATP content and decreased the ADP and AMP content in the intestinal tissue, inhibited the low expression of occludin between the ileum epithelial cells, and protected the intestinal mucosal barrier. In addition, the SNS treatment inhibited the increase of ECs and MCs in the ileum and the increase of 5-HT content in plasma and the ileum.

Previous studies have demonstrated that chronic stress activated DβH and c-fos in rat brain locus coeruleus (Sun et al., 2017), which promoted intestinal peristalsis and smooth muscle contraction (Kurahashi et al., 2020). However, whether the activation of locus coeruleus would also result in intestinal artery contractions is not known. In the present study, we found that chronic stress could also cause intestinal artery contractions through an increased release of NE and the activation of α1-ARs and the downstream MLCK-MLC pathway, which were all reversed by the SNS treatment.

It has been previously studied that chronic stress led to a low occludin expression between the ileum epithelial cells, which is an important marker of the mucosal barrier damage (Kuo et al., 2019; Rawat et al., 2020). Occludin is maintained by cytoskeleton fibrous actin, which is assembled using ATP (Han et al., 2017). However, the effect of chronic stress on intestinal energy metabolism is unclear. In this study, we found that chronic stress induced intestinal artery contractions, leading to ischemia, hypoxia, decreased ATP5D expression and ATP production, and low occludin expression. This phenomenon was ameliorated by the SNS treatment, probably by inhibiting the contraction of small arteries, improving blood and oxygen supply, increased ATP5D expression and ATP production, thereby restoring the cytoskeleton, occludin expression, and the mucosal barrier integrity.

The locus coeruleus is a norepinephrinergic neuronal nucleus in the brain that synthesizes and releases NE. Our previous study has demonstrated that chronic unpredictable mild stress can cause high expressions of DβH and c-fos in the rat locus coeruleus and increased NE content in plasma and follicular cells (Sun et al., 2017). However, there is no effective means to regulate its activity. The present study found that SNS inhibited the chronic stress-induced high expression of DβH and c-fos in the locus coeruleus, and reduced plasma and intestinal NE, which may contribute to its inhibitory effect on chronic stress-induced diarrhea.

ECs and MCs are the main cells responsible for the secretion of 5-HT upon activation. 5-HT could cause intestine and mesenteric artery contractions (Spiller, 2002; Sung et al., 2013) and promote mucus secretion (Camilleri, 2009). Clinical studies suggest that 5-HT levels, and EC and MC numbers are increased in patients with IBS-D (Dunlop et al., 2003; Sikander et al., 2009). We also found that SNS could inhibit the increase of ECs and MCs in the ileum, and plasma and intestinal 5-HT levels (Gershon, 2013; Bellono et al., 2017; Mukai et al., 2018), which may contribute to its inhibition on the symptoms of chronic stress-induced diarrhea.

The limitation of our study is that, it only demonstrated the effect of SNS on diarrhea caused by chronic restraint stress *in vivo*. However, the effect of the active ingredients of SNS on intestinal epithelial cells needs to be further explored in the future.

In conclusion, the present study demonstrated that SNS could improve CRS-induced diarrhea in rats, which was mediated by inhibition of the locus coeruleus activation, NE release, intestinal artery contractions, intestinal mucosal barrier damage, intestinal energy metabolism abnormality, increased ECs, MCs, and 5-HT secretion. These results provided experimental evidence supporting the clinical usage of SNS for the treatment of diarrhea caused by chronic stress.

## Data Availability

The original contributions presented in the study are included in the article/supplementary material; further inquiries can be directed to the corresponding authors.

## References

[B1] Aguilera-Lizarraga J. Hussein H. Boeckxstaens G. E. (2022). Immune activation in irritable bowel syndrome: What is the evidence? Nat. Rev. Immunol.. 10.1038/s41577-022-00700-9 35296814

[B2] Bellono N. W. Bayrer J. R. Leitch D. B. Castro J. Zhang C. O'Donnell T. A. (2017). Enterochromaffin cells are gut chemosensors that couple to sensory neural pathways. Cell. 170 (1), 185–198. e16. 10.1016/j.cell.2017.05.034 28648659 PMC5839326

[B3] Camilleri M. (2009). Serotonin in the gastrointestinal tract. Curr. Opin. Endocrinol. Diabetes Obes. 16 (1), 53–59. 10.1097/med.0b013e32831e9c8e 19115522 PMC2694720

[B4] Carrasco-Labra A. Lytvyn L. Falck-Ytter Y. Surawicz C. M. Chey W. D. (2019). AGA technical review on the evaluation of functional diarrhea and diarrhea-predominant irritable bowel syndrome in adults (IBS-D). Gastroenterology 157 (3), 859–880. 10.1053/j.gastro.2019.06.014 31351880

[B5] Castellani J. W. Young A. J. (2016). Human physiological responses to cold exposure: Acute responses and acclimatization to prolonged exposure. Auton. Neurosci. 196, 63–74. 10.1016/j.autneu.2016.02.009 26924539

[B6] Chang L. (2021). How to approach a patient with difficult-to-treat IBS. Gastroenterology 161 (4), 1092–1098.e3. e3. 10.1053/j.gastro.2021.07.034 34331916

[B7] Chang X. Zhao L. Wang J. Lu X. Zhang S. (2017). Sini-san improves duodenal tight junction integrity in a rat model of functional dyspepsia. BMC Complement. Altern. Med. 17 (1), 432. 10.1186/s12906-017-1938-2 28854971 PMC5577804

[B8] Dunlop S. P. Jenkins D. Neal K. R. Spiller R. C. (2003). Relative importance of enterochromaffin cell hyperplasia, anxiety, and depression in postinfectious IBS. Gastroenterology 125 (6), 1651–1659. 10.1053/j.gastro.2003.09.028 14724817

[B9] Ek W. E. Reznichenko A. Ripke S. Niesler B. Zucchelli M. Rivera N. V. (2015). Exploring the genetics of irritable bowel syndrome: A GWA study in the general population and replication in multinational case-control cohorts. Gut 64 (11), 1774–1782. 10.1136/gutjnl-2014-307997 25248455

[B10] Gao J. Xiong T. Grabauskas G. Owyang C. (2022). Mucosal serotonin reuptake transporter expression in irritable bowel syndrome is modulated by gut microbiota via mast cell-prostaglandin E2. Gastroenterology 162, 1962–1974.e6. 10.1053/j.gastro.2022.02.016 35167867 PMC9117493

[B11] Gershon M. D. (2013). 5-Hydroxytryptamine (serotonin) in the gastrointestinal tract. Curr. Opin. Endocrinol. Diabetes Obes. 20 (1), 14–21. 10.1097/MED.0b013e32835bc703 23222853 PMC3708472

[B12] Han J. Y. Li Q. Ma Z. Z. Fan J. Y. (2017). Effects and mechanisms of compound Chinese medicine and major ingredients on microcirculatory dysfunction and organ injury induced by ischemia/reperfusion. Pharmacol. Ther. 177, 146–173. 10.1016/j.pharmthera.2017.03.005 28322971

[B13] Jiang J. Chen Y. Hu Z. Li H. Ye J. Yu Z. (2021). Effectiveness of tong-xie-yao-fang combined with Si-Ni-san for irritable bowel syndrome: A protocol for systematic review and meta-analysis. Med. Baltim. 100 (11), e25198. 10.1097/MD.0000000000025198 PMC7982163 33726014

[B14] Julio-Pieper M. O'Mahony C. M. Clarke G. Bravo J. A. Dinan T. G. Cryan J. F. (2012). Chronic stress-induced alterations in mouse colonic 5-HT and defecation responses are strain dependent. Stress 15 (2), 218–226. 10.3109/10253890.2011.607524 21875301

[B15] Jun H. Ko S. J. Kim K. Kim J. Park J. W. (2022). An overview of systematic reviews of herbal medicine for irritable bowel syndrome. Front. Pharmacol. 13, 894122. 10.3389/fphar.2022.894122 35662700 PMC9158123

[B16] Kawano K. Mori T. Fu L. Ito T. Niisato T. Yoshida S. (2005). Comparison between partial agonist (ME3412) and antagonist (alosetron) of 5-hydroxytryptamine 3 receptor on gastrointestinal function. Neurogastroenterol. Motil. 17 (2), 290–301. 10.1111/j.1365-2982.2004.00622.x 15787949

[B17] Kuo W. T. Shen L. Zuo L. Shashikanth N. Ong M. Wu L. (2019). Inflammation-induced occludin downregulation limits epithelial apoptosis by suppressing caspase-3 expression. Gastroenterology 157 (5), 1323–1337. 10.1053/j.gastro.2019.07.058 31401143 PMC6815722

[B18] Kurahashi M. Kito Y. Hara M. Takeyama H. Sanders K. M. Hashitani H. (2020). Norepinephrine has dual effects on human colonic contractions through distinct subtypes of alpha 1 adrenoceptors. Cell. Mol. Gastroenterol. Hepatol. 10 (3), 658–671. e1. 10.1016/j.jcmgh.2020.04.015 32376421 PMC7474159

[B19] Li B. Rui J. Ding X. Chen Y. Yang X. (2019). Deciphering the multicomponent synergy mechanisms of SiNiSan prescription on irritable bowel syndrome using a bioinformatics/network topology based strategy. Phytomedicine 63, 152982. 10.1016/j.phymed.2019.152982 31299593

[B20] Li X. Liu Q. Yu J. Zhang R. Sun T. Jiang W. (2021). Costunolide ameliorates intestinal dysfunction and depressive behaviour in mice with stress-induced irritable bowel syndrome via colonic mast cell activation and central 5-hydroxytryptamine metabolism. Food Funct. 12 (9), 4142–4151. 10.1039/d0fo03340e 33977961

[B21] Linan-Rico A. Ochoa-Cortes F. Beyder A. Soghomonyan S. Zuleta-Alarcon A. Coppola V. (2016). Mechanosensory signaling in enterochromaffin cells and 5-HT release: Potential implications for gut inflammation. Front. Neurosci. 10, 564. 10.3389/fnins.2016.00564 28066160 PMC5165017

[B22] Long Y. Huang Z. Deng Y. Chu H. Zheng X. Yang J. (2017). Prevalence and risk factors for functional bowel disorders in south China: A population based study using the rome III criteria. Neurogastroenterol. Motil. 29 (1), e12897. 10.1111/nmo.12897 27412422

[B23] Lovell R. M. Ford A. C. (2012). Global prevalence of and risk factors for irritable bowel syndrome: A meta-analysis. Clin. Gastroenterol. Hepatol. 10 (7), 712–721. e4. 10.1016/j.cgh.2012.02.029 22426087

[B24] Mukai K. Tsai M. Saito H. Galli S. J. (2018). Mast cells as sources of cytokines, chemokines, and growth factors. Immunol. Rev. 282 (1), 121–150. 10.1111/imr.12634 29431212 PMC5813811

[B25] Peery A. F. Crockett S. D. Murphy C. C. Jensen E. T. Kim H. P. Egberg M. D. (2022). Burden and cost of gastrointestinal, liver, and pancreatic diseases in the United States: Update 2021. Gastroenterology 162 (2), 621–644. 10.1053/j.gastro.2021.10.017 34678215 PMC10756322

[B26] Pei L. Geng H. Guo J. Yang G. Wang L. Shen R. (2020). Effect of acupuncture in patients with irritable bowel syndrome: A randomized controlled trial. Mayo Clin. Proc. 95 (8), 1671–1683. 10.1016/j.mayocp.2020.01.042 32499125

[B27] Pohl C. S. Medland J. E. Mackey E. Edwards L. L. Bagley K. D. Dewilde M. P. (2017). Early weaning stress induces chronic functional diarrhea, intestinal barrier defects, and increased mast cell activity in a porcine model of early life adversity. Neurogastroenterol. Motil. 29 (11), e13118. 10.1111/nmo.13118 PMC5650513 28573751

[B28] Rawat M. Nighot M. Al-Sadi R. Gupta Y. Viszwapriya D. Yochum G. (2020). IL1B increases intestinal tight junction permeability by up-regulation of MIR200C-3p, which degrades occludin mRNA. Gastroenterology 159 (4), 1375–1389. 10.1053/j.gastro.2020.06.038 32569770 PMC11752806

[B29] Sikander A. Rana S. V. Prasad K. K. (2009). Role of serotonin in gastrointestinal motility and irritable bowel syndrome. Clin. Chim. Acta. 403 (1-2), 47–55. 10.1016/j.cca.2009.01.028 19361459

[B30] Sperber A. D. Dumitrascu D. Fukudo S. Gerson C. Ghoshal U. C. Gwee K. A. (2017). The global prevalence of IBS in adults remains elusive due to the heterogeneity of studies: A rome foundation working team literature review. Gut 66 (6), 1075–1082. 10.1136/gutjnl-2015-311240 26818616

[B31] Spiller R. (2002). Serotonergic modulating drugs for functional gastrointestinal diseases. Br. J. Clin. Pharmacol. 54 (1), 11–20. 10.1046/j.1365-2125.2002.01612.x 12100220 PMC1874383

[B32] Sun H. Y. Li Q. Liu Y. Y. Wei X. H. Pan C. S. Fan J. Y. (2017). Xiao-Yao-San, a Chinese medicine formula, ameliorates chronic unpredictable mild stress induced polycystic ovary in rat. Front. Physiol. 8, 729. 10.3389/fphys.2017.00729 29018356 PMC5614964

[B33] Sung D. J. Noh H. J. Kim J. G. Park S. W. Kim B. Cho H. (2013). Serotonin contracts the rat mesenteric artery by inhibiting 4-aminopyridine-sensitive Kv channels via the 5-HT2A receptor and Src tyrosine kinase. Exp. Mol. Med. 45, e67. 10.1038/emm.2013.116 24336234 PMC3880459

[B34] Wang Z. Xu M. Shi Z. Bao C. Liu H. Zhou C. (2022). Mild moxibustion for irritable bowel syndrome with diarrhea (IBS-D): A randomized controlled trial. J. Ethnopharmacol. 289, 115064. 10.1016/j.jep.2022.115064 35114338

[B35] Wong H. Qin H. Y. Tsang S. W. Zuo X. Che S. Chow C. (2019). Early life stress disrupts intestinal homeostasis via NGF-TrkA signaling. Nat. Commun. 10 (1), 1745. 10.1038/s41467-019-09744-3 30988299 PMC6465335

[B36] Zhang H. Liu J. Qu D. Wang L. Wong C. M. Lau C. W. (2018). Serum exosomes mediate delivery of arginase 1 as a novel mechanism for endothelial dysfunction in diabetes. Proc. Natl. Acad. Sci. U. S. A. 115 (29), E6927-E6936. 10.1073/pnas.1721521115 29967177 PMC6055191

[B37] Zhu C. Zhao L. Zhao J. Zhang S. (2020). Sini San ameliorates duodenal mucosal barrier injury and lowgrade inflammation via the CRF pathway in a rat model of functional dyspepsia. Int. J. Mol. Med. 45 (1), 53–60. 10.3892/ijmm.2019.4394 31746413 PMC6889936

